# Neutrophil Extracellular DNA Traps Induce Autoantigen Production by Airway Epithelial Cells

**DOI:** 10.1155/2017/5675029

**Published:** 2017-08-30

**Authors:** Youngwoo Choi, Le Duy Pham, Dong-Hyun Lee, Ga-Young Ban, Ji-Ho Lee, Seung-Hyun Kim, Hae-Sim Park

**Affiliations:** ^1^Department of Allergy and Clinical Immunology, Ajou University School of Medicine, Suwon, Republic of Korea; ^2^Department of Biomedical Sciences, Graduate School of Ajou University, Suwon, Republic of Korea; ^3^Faculty of Medicine, University of Medicine and Pharmacy, Ho Chi Minh City, Vietnam; ^4^Clinical Trial Center, Ajou University Medical Center, Suwon, Republic of Korea

## Abstract

The hypothesis of autoimmune involvement in asthma has received much recent interest. Autoantibodies, such as anti-cytokeratin (CK) 18, anti-CK19, and anti-*α*-enolase antibodies, react with self-antigens and are found at high levels in the sera of patients with severe asthma (SA). However, the mechanisms underlying autoantibody production in SA have not been fully determined. The present study was conducted to demonstrate that neutrophil extracellular DNA traps (NETs), cytotoxic molecules released from neutrophils, are a key player in the stimulation of airway epithelial cells (AECs) to produce autoantigens. This study showed that NETs significantly increased the intracellular expression of tissue transglutaminase (tTG) but did not affect that of CK18 in AECs. NETs induced the extracellular release of both tTG and CK18 in a concentration-dependent manner. Moreover, NETs directly degraded intracellular *α*-enolase into small fragments. However, antibodies against neutrophil elastase (NE) or myeloperoxidase (MPO) attenuated the effects of NETs on AECs. Furthermore, each NET isolated from healthy controls (HC), nonsevere asthma (NSA), and SA had different characteristics. Taken together, these findings suggest that AECs exposed to NETs may exhibit higher autoantigen production, especially in SA. Therefore, targeting of NETs may represent a new therapy for neutrophilic asthma with a high level of autoantigens.

## 1. Introduction

Asthma is a chronic inflammatory disease of the airways. Approximately 5%–10% of patients with asthma exhibit severe symptoms that are not easily controlled by regular medication [1–3]. Severe neutrophilic asthma is a major phenotype of severe asthma (SA), in which neutrophils significantly contribute to the exacerbation of symptoms and airway remodeling [4]. However, the role of neutrophils in pathophysiological mechanisms responsible for SA has not been fully determined.

Recent studies have demonstrated that neutrophils participate in autoimmune disease [5, 6]; furthermore, autoimmune mechanisms, such as the deposition of autoantibodies in specific tissues, are known to play a role in asthma [7]. Our group previously found circulating autoantibodies, such as anti-cytokeratin (CK) 18, anti-CK19, and anti-*α*-enolase antibodies, against proteins expressed by airway epithelial cells (AECs) in patients with SA [8, 9]. Moreover, antibodies against tissue transglutaminase (tTG) were detected in patients with toluene diisocyanate-induced occupational asthma, which is generally associated with a neutrophilic phenotype [10]. Although autoimmune responses are associated with the pathogenesis of asthma [11], the mechanisms by which these autoantigens are generated in SA remain poorly understood.

Neutrophils, which are the most abundant leukocytes in humans, produce cytotoxic granule proteins [12]. Recently, it has been suggested that activated neutrophils undergo a novel form of cell death during which a meshwork of chromatin with bound granule proteins, known as neutrophil extracellular DNA traps (NETs), is released [13, 14]. A previous study demonstrated high levels of NETs in patients with SA and showed that NETs stimulate the production of proinflammatory cytokines by AECs [15]. In addition, NETs have been suggested to elicit the production of autoantibodies in various autoimmune diseases [16]. However, the mechanisms by which high levels of NETs induce autoantigen production in SA have not been demonstrated to date.

Diverse proteins have been identified as airway epithelial autoantigens associated with SA [8]. To investigate the effects of NETs on AECs to produce autoantigens, the present study attempted to evaluate protein expression, especially that of CK18, tTG, and *α*-enolase, in these cells.

## 2. Materials and Methods

### 2.1. Study Subjects

The study was approved by the Institutional Review Board of Ajou University Hospital (AJIRB-GEN-GEN-09-140). All patients provided written informed consent at the time of recruitment. We enrolled 5 patients with SA who were diagnosed as a result of recurrent episodes of wheezing, dyspnea, cough, and evidence of either airway hyperresponsiveness to methacholine or reversible airway obstruction improved by treatment with a short-acting *β*_2_-agonist [17]. To investigate differences in NETs individually, 3 healthy controls (HC), individuals with nonsevere asthma (NSA), and individuals with SA were recruited, respectively.

### 2.2. Isolation of Neutrophils from Peripheral Blood

At the time of diagnosis, venous blood from patients with asthma was collected into BD Vacutainer tubes containing acid citrate dextrose solution (BD Biosciences, Franklin Lakes, NJ, USA) and processed within 2 h of collection. Blood was layered on Lymphoprep solution (Axis-Shield, Oslo, Norway), followed by centrifugation at 879 ×g at 20°C for 25 min, without any brake. The layer containing granulocytes was separated and placed in Hank's balanced salt solution (HBSS) buffer, with 2 mM ethylenediaminetetraacetic acid (EDTA) and 2% dextran, for 20 min at 26–30°C. The neutrophil-rich layer was collected and washed once with HBSS buffer containing 2 mM EDTA. Red blood cells (RBCs) were eliminated by hypotonic lysis. Peripheral blood neutrophils were maintained in RPMI-1640 medium with 2% fetal bovine serum (FBS), penicillin (100 IU/mL), and streptomycin (50 *μ*g/mL).

### 2.3. Induction of Neutrophil Extracellular DNA Traps

Peripheral blood neutrophils were stimulated and isolated as described previously [18]. Isolated neutrophils were treated with 100 nM phorbol myristate acetate (PMA) for 3 h. Each well was washed twice with RPMI to eliminate PMA and NET-dissociated molecules. To confirm the removal of residual PMA, which could affect target cells, from isolated NETs, the supernatants from the third wash (Sup) were collected and used as controls to treat target cells. RPMI (1 mL) containing micrococcal nuclease (MNase) (1 U/*μ*L) was then added to each well to digest NETs at 37°C for 20 min. NETs were collected by eliminating cell or cell debris. Isolated NETs were quantified by measuring the DNA concentration using the PicoGreen assay (Invitrogen, Paisley, UK) according to the manufacturer's instructions.

### 2.4. Detection of Neutrophil Extracellular DNA Traps

Peripheral blood neutrophils (2 × 10^5^) were seeded on L-lysine-coated slides (Polysciences, Warrington, PA, USA). Neutrophils were stained with anti-NE antibody and DAPI to detect NET formation. Cells were examined under a Zeiss LSM710 confocal microscope with a 63 × oil objective lens (Carl Zeiss, Oberkochen, Germany). The images were analyzed using ZEN 2009 software (Carl Zeiss).

### 2.5. Cell Culture

A549 cells (American Type Culture Collection, Manassas, VA, USA) were cultured in RPMI 1640 Medium supplemented with 10% FBS, penicillin (100 IU/mL), and streptomycin (50 *μ*g/mL). Cells were grown at 37°C in humidified air with 5% CO_2_. To investigate autoantigen production, cells were treated with isolated NETs with final DNA concentrations of 1 *μ*g/mL and 5 *μ*g/mL. To investigate the effects of neutrophil elastase (NE) and myeloperoxidase (MPO), cells were treated with NETs that had been preincubated with antibodies against NE (Santa Cruz, Dallas, TX, USA) or MPO (Cell Signaling, Minneapolis, MN, USA).

### 2.6. Immunoblot Analysis

CK18, tTG, and *α*-enolase expressions in cell lysates and culture supernatants were evaluated by Western blot analysis. Consequently, relative expression of each protein to actin was evaluated. Anti-CK18 antibody (Cell Signaling), anti-tTG (Cell Signaling) antibody, anti-*α*-enolase antibody (Santa Cruz), and anti-actin antibody (Santa Cruz) were used.

### 2.7. Statistical Analysis

Data were analyzed by one-way ANOVA with Bonferroni's post hoc test. Statistical analyses were performed with SPSS software, version 22.0 (SPSS Inc., Chicago, IL, USA). *P* < 0.05 was considered to indicate statistical significance. GRAPHPAD PRISM 5.0 software (GraphPad Inc., San Diego, CA, USA) was used for graphs, with values presented as the mean ± standard deviation (SD) of at least three independent experiments.

## 3. Results

### 3.1. Clinical Characteristics of the Study Subjects

Five patients with SA (GINA guidelines step 4-5) were enrolled for isolation of NETs. Three males and two females were recruited; the mean age of the patients was 28.60 ± 6.66 years. The baseline % forced expiratory volume in 1 sec (FEV_1_) was 99.02 ± 14.18%. All females, but none of the males, were atopic.

### 3.2. Activated Peripheral Blood Neutrophils Release Neutrophil Extracellular DNA Traps

**Figure 1:**
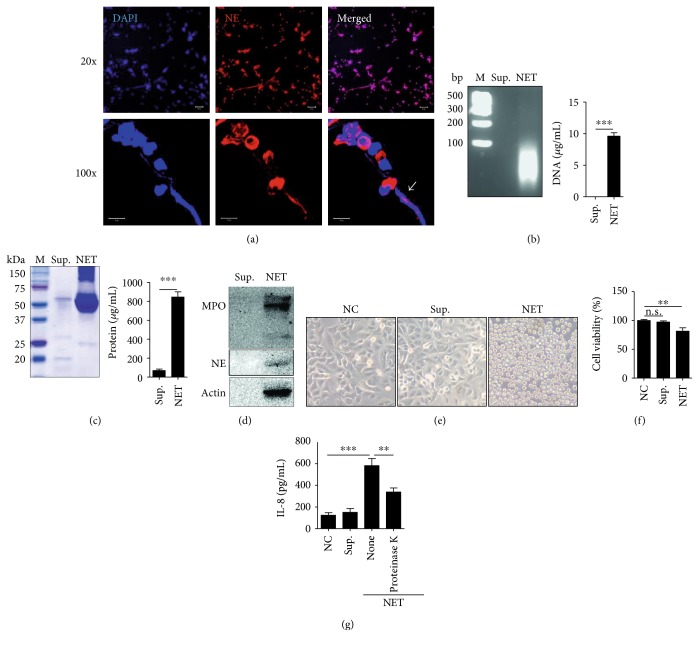
Characterization of NETs isolated from peripheral blood neutrophils of SA. (a) Detection of NET production (a white arrow); scale bar, 10 *μ*m. (b) DNA bands (left panel) and concentration (right panel). (c) Protein profile (left panel) and concentration (right panel). (d) Western blot analysis of granule proteins. (e) Change in A549 cell morphology following NET treatment. (f) Cell viability assessed by Cell Counting Kit-8 (CCK8) assay. (g) Proinflammatory effects of NETs on A549 cells. ^∗∗^*P* < 0.01, ^∗∗∗^*P* < 0.001. n.s., not significant.

Peripheral blood neutrophils stimulated with phorbol myristate acetate (PMA) produced not only web-like extracellular DNA but also cytotoxic granule proteins such as neutrophil elastase (NE). Blue DAPI staining indicates the nucleus (in particular, DNA) and red colored staining with anti-NE antibody indicates NE. Neutrophils stained with both dyes are activated cells that undergo cell death following NET production (Figure 1(a)).

### 3.3. Neutrophil Extracellular DNA Traps Contain Specific Extracellular DNA and Granule Proteins

To investigate extracellular DNA released by neutrophils, NETs were loaded on 0.8% agarose gel. NETs treated with micrococcal nuclease (MNase) showed specific DNA bands of under 100 bp in size (Figure 1(b), left panel). The DNA concentration was approximately 10 *μ*g/mL (Figure 1(b), right panel). Protein profile analysis performed by Coomassie Brilliant Blue staining indicated that proteins in NETs were of a specific size (between 50 and 75 kDa) (Figure 1(c), left panel). The protein concentration was approximately 800 *μ*g/mL (Figure 1(c), right panel). Western blot analysis of NETs showed that granule proteins colocalize with DNA (Figure 1(d)).

### 3.4. Neutrophil Extracellular DNA Traps Exert Cytotoxic Effects on AECs to Induce Inflammation

To demonstrate the cytotoxic effects of NETs on AECs, cell morphology was observed following NET treatment. Initially, AECs that were elongated and spindle-shaped were observed to gently attach to culture plates. However, the cells were found to acquire a round shape and detach from the culture plates after NET treatment (Figure 1(e)). Cell viability was also measured; NETs at a final concentration of 5 *μ*g/mL DNA induced more than 30% cell death in the total cell population (Figure 1(f)). When AECs were treated with NETs, the cells produced significantly high levels of IL-8. However, NETs preincubated with proteinase K elicited a lower degree of production of proinflammatory cytokines by AECs (Figure 1(g)).

### 3.5. Ability of Neutrophil Extracellular DNA Traps to Induce CK18 Production from AECs

**Figure 2:**
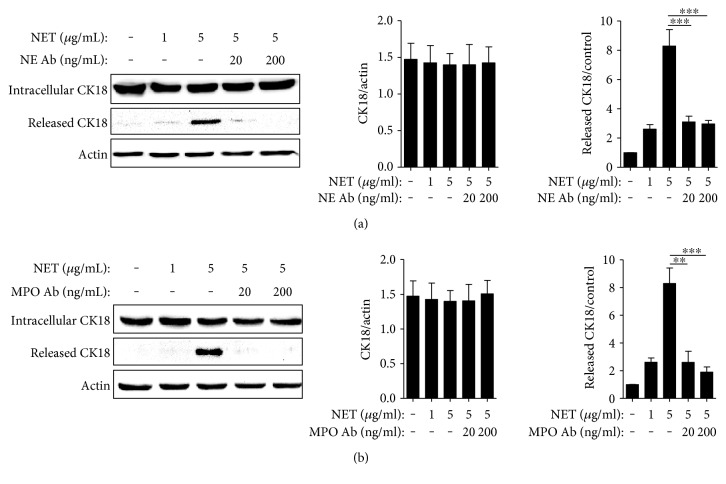
NETs induced CK18 expression and extracellular release from A549 cells. Effects of NETs on A549 cells incubated with/without NE (a) or MPO (b) antibody. Significance is represented by ^∗∗^*P* < 0.01 and ^∗∗∗^*P* < 0.001.

To determine whether NETs could enhance autoantigen production, CK18 expression in cell lysates and culture supernatants from AECs was evaluated by Western blot analysis. NETs were found to significantly upregulate the release of CK18 into the culture supernatant in a concentration-dependent manner. However, intracellular expression of CK18 was not affected by NETs. NET preincubated with antibodies against NE or MPO showed weaker effects on AECs (Figures 2(a) and 2(b)). We confirmed that NETs, at the concentrations tested, did not contain a detectable amount (if any) of CK18.

### 3.6. Expression of tTG in AECs Is Mediated by Neutrophil Extracellular DNA Traps

**Figure 3:**
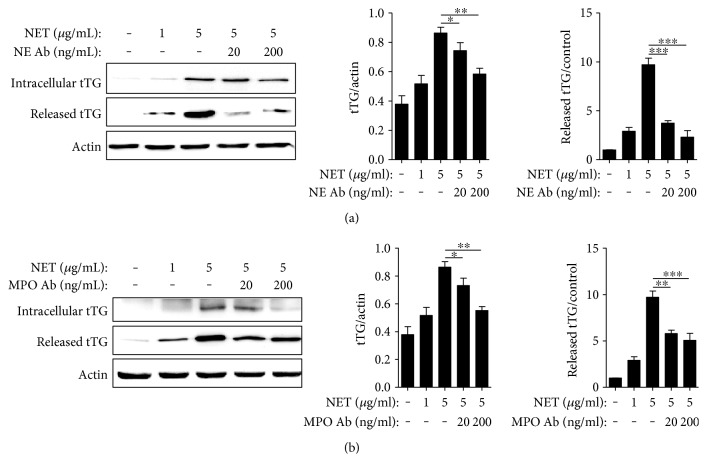
NETs induced tTG expression and extracellular release from A549 cells. Effects of NETs on A549 cells incubated with or without NE (a) or MPO (b) antibody. Significance is represented by ^∗^*P* < 0.05, ^∗∗^*P* < 0.01, and ^∗∗∗^*P* < 0.001.

Another autoantigen, tTG, was detected in both cell lysates and culture supernatants. In contrast to the expression of CK18, NETs dramatically increased the intracellular expression of tTG. In addition, NETs also concentration-dependently induced the release of CK18 into the culture supernatant. Similar to the CK18 expression data, NETs preincubated with antibodies against NE or MPO had attenuated effects on AECs in terms of eliciting intracellular tTG expression and extracellular tTG release (Figures 3(a) and 3(b)).

### 3.7. Neutrophil Extracellular DNA Traps Degrade Intracellular *α*-Enolase into Small Fragments

**Figure 4:**
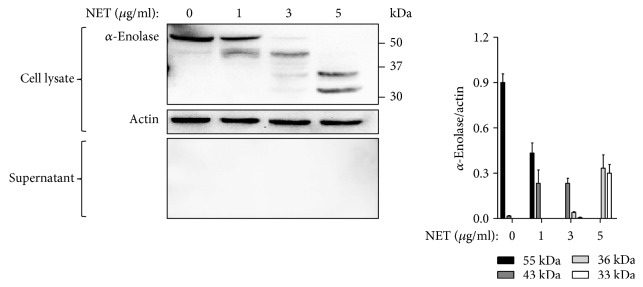
*α*-Enolase in A549 cells was degraded into small fragments by NET treatment. *α*-Enolase expression in cell lysates and culture supernatants was evaluated by Western blot.

The expression of *α*-enolase in AECs following NET treatment was investigated. NETs degraded intracellular *α*-enolase (55 kDa) into a 43-kDa fragment at a concentration of 1 *μ*g/mL DNA, or 43-kDa and 36-kDa fragments at 3 *μ*g/mL of DNA, and 36-kDa and 32-kDa fragments at 5 *μ*g/mL DNA. However, neither *α*-enolase nor its fragments were detected in the cell culture supernatants (Figure 4).

### 3.8. Different Characteristics of Neutrophil Extracellular DNA Traps Isolated from HC, NSA, and SA

**Figure 5:**
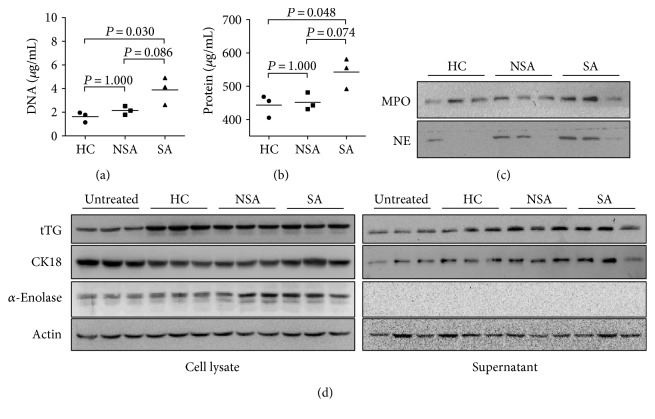
Comparison of NETs isolated from HC, NSA, and SA. (a) DNA concentration measured by PicoGreen assay. (b) Protein concentration evaluated by BCA assay. (c) Western blot analysis of NE and MPO in NETs. (d) Change in protein expression of AECs by NET treatment.

HC, NSA, and SA patients were enrolled, respectively, to identify differences in each NET of the study subjects (in Supplementary Table available online at https://doi.org/10.1155/2017/5675029). NETs extracted from SA had higher concentration of DNA compared to those from HC and NSA (Figure 5(a)). These NETs also contained more proteins (Figure 5(b)). In addition, the composition of granule proteins in each NET was different (Figure 5(c)). Furthermore, every NET showed significant effects on protein expression in AECs (Figure 5(d)).

## 4. Discussion

Neutrophil activity has been implicated in SA; however, the precise role of neutrophils remains unclear [4]. A recent study demonstrated that activated neutrophils induce NETs in patients with SA, thus activating eosinophils and AECs and enhancing airway inflammation [15]. This study proposes another role of neutrophils in SA: the production of NETs, which could increase autoantigen production by AECs. Autoimmune responses to such autoantigens may represent a pathogenic mechanism underlying the induction of airway inflammation.

The cytotoxic effects of NETs may contribute to the pathogenesis of asthma [19]. NE and MPO are the two main granule proteins localized within NETs that are implicated in airway epithelium and cell damage [20, 21]. MPO has been believed to play a more critical role in this process [18]. However, in the current study, blocking the exposure of these two granular proteins by preincubation with antibodies against NE or MPO resulted in inhibitory effects on AECs, thereby demonstrating that both proteins play an equally important role. Moreover, NETs preincubated with proteinase K showed reduced toxicity. The present study suggests that airway inflammation in asthma may be induced by both extracellular DNA and granule proteins in NETs.

NETs are also known to play a role in autoimmune disease [16]; the induction of such responses is considered to contribute to asthma. Autoantibodies have been suggested to directly or indirectly (via T cell interactions) induce cytotoxicity, thereby enhancing airway inflammation in SA [22]. Our group previously detected several autoantibodies, such as anti-CK18, anti-CK19, anti-*α*-enolase, and tTG antibodies, in the sera of patients with SA [10, 23–25]. However, the mechanisms underlying autoantibody production in SA are not clear. The present study showed that NET-treated AECs significantly increased the expression of CK18 and tTG. Moreover, NETs degraded intracellular *α*-enolase into several small fragments, which may have been comprised of autoantigens, to elicit autoantibody production. The current study proposes a new pathway for enhancing autoantibody production in SA, through the production or modification of autoantigens by NETs.

Neutrophils from patients with SA and NSA release different amounts of NETs; however, a significantly higher release was noted in SA patients [15]. The previous study did not perform a detailed characterization of NETs individually. This study showed different compositions of each NET, even within the same group. However, all NETs isolated from neutrophils affected protein expression in AECs. As neutrophils from SA produced a large number of NETs, the degree of neutrophil activation could be one possibility that enhances autoantigen production and increases asthma severity.

The present study has limitations: First, a positive correlation between autoantigen and autoantibody production *in vivo*, which would have directly explained the increased levels of autoantibodies, was not demonstrated. Secondly, although NETs exert cytotoxic effects, resulting in the production and modification of intracellular autoantigens, it is not clear why the expression of each autoantigen is different in AECs. This may be attributed to the complexity of the mechanisms by which NETs affect signaling molecules in the cells. Thirdly, the potential contribution of other immune cells to autoimmune responses involving NETs should be additionally clarified.

## 5. Conclusions

In conclusion, activated neutrophils produce NETs, which could contribute to airway epithelial damage, proinflammatory cytokine induction, and autoantigen production. Therefore, inhibition of NETs may be a novel therapeutic approach to asthma presenting a neutrophilic phenotype.

## Supplementary Material

Supplementary Table. Demographic data of the study subjects.
