# Supplementary material for: Neutrophil Extracellular DNA Traps Induce Autoantigen Production by Airway Epithelial Cells
Source: Mediators Inflamm. 2017 Aug 30;2017:5675029. doi: 10.1155/2017/5675029 (PMC5603142; doi:10.1155/2017/5675029)
Supplement: Supplementary file 1 — Supplementary Table. Demographic data of the study subjects. [file 5675029.f1.pdf]

Supplementary Information

Neutrophil extracellular DNA traps induce autoantigen production by airway epithelial cells

Youngwoo Choi, PhD,[1*] Duy Le Pham, MD, PhD,[1,2,4*] Dong-Hyun Lee, MS,[2] Ga-Young Ban, MD, PhD,[1] Ji-Ho Lee, MD,[1] Seung-Hyun Kim, PhD,[3] Hae-Sim Park, MD, PhD[1,2,3]

[1]Department of Allergy and Clinical Immunology; [2]Department of Biomedical Sciences; [3]Clinical Trial Center, Ajou University Medical Center, Suwon, South Korea; [4]Medicine Faculty, University of Medicine and Pharmacy, Ho Chi Minh city, Viet Nam.

*These authors contributed equally to this work.

Corresponding author: Professor Hae-Sim Park, MD, PhD

Supplementary Table. Demographic data of the study subjects.

	HC (n=3)	NSA (n=3)	SA (n=3)
Age (y)	31.00 ± 4.36	53.55 ± 22.03	60.00 ± 7.81
Female sex (%)	100.00	66.67	66.67
Atopy (%)	66.67	100.00	66.67
FEV_1 (%)	NA	95.23 ± 15.34	69.87 ± 15.87
TEC (/μL)	NA	196.67 ± 158.85	643.74 ± 371.67
Serum IgE (kU/L)	NA	129.67 ± 68.30	220.67 ± 170.41
Sputum neutrophils (%)	NA	44.00 ± 57.98	69.67 ± 27.39

FEV_1, forced expiratory volume in 1 s; TEC, total eosinophil count; IgE, immunoglobulin E; NA, not applicable.
